# Hypolipidemic effect of β-caryophyllene in high fat diet and fructose induced type-2 diabetic adult male rats

**DOI:** 10.6026/973206300181087

**Published:** 2022-11-30

**Authors:** Vadivel Mani, Manikandan Balraj, Nithya Rajapandian, Chaitra Jinka, Shyamaladevi Babu

**Affiliations:** 1 Department of Biochemistry, Arunai Medical College and Hospital, Tiruvanamallai- 606603, Tamilnadu, India; 2 Department of Physiology, Konaseema Institute of Medical Sciences and Research Foundation, Amalapuram, East Godavari Dt-533201, Andhra Pradesh, India; 3 Department of Physiology, Arunai Medical College and Hospital, Tiruvanamallai- 606603, Tamilnadu, India; 4 Syngene International Limited, Biocon park, Bangalore, Karnataka, India; 5 Faculty of Allied Health Sciences, Chettinad Hospital and Research Institute, Chettinad Academy of Research and Education, Kelambakkam - 603103, Tamil Nadu, India

**Keywords:** Type 2 diabetes, β-caryophyllene, high fat diet, skeletal muscle, glucose uptake, glycogen

## Abstract

Skeletal muscle is responsible for the majority of insulin-stimulated whole-body glucose elimination, under normal circumstances. High dietary fat consumption increases stored fat mass and is a major risk factor for metabolic disorders. The conventional pharmacological treatments are associated with many adverse side effects and high rates of secondary failure which lead to an increasing demand for natural products with anti-diabetic activity and lesser side effects. β-Caryophyllene is a naturally occurring sequiterpene that may be found in cannabis and a range of culinary herbs and spices. It contains antioxidant, anti-inflammatory, and anti-lipidemic effects, among others. However, the effect of β-Caryophyllene on glucose absorption and oxidation, is yet unknown. Hence, the current study was intended to investigate the anti-diabetic impact of β-Caryophyllene in type-2 diabetes caused by a high-fat diet. To evaluate its anti-diabetic efficacy, high fat diet and fructose-induced type-2 diabetic rats were administered an effective dosage of β-Caryophyllene (200 mg/kg b.wt, orally for 30 days). The treatment of diabetes-induced rats with β-Caryophyllene restored the altered levels of blood glucose, serum insulin as well as lipid parameters. Our findings show that β-caryophyllene improves glycemia control by alleviating dyslipidemia in type-2 diabetic rats. From the present findings, it is evident that β-caryophyllene can be used as an anti-diabetic drug.

## Background:

Diabetes mellitus is a chronic metabolic disorder identified by inadequate insulin secretion, or resistance to insulin action, or both. It is associated with disturbances in carbohydrate, lipid and protein metabolism, which leads to hyperglycemia, hyperlipidemia, hyper insulinemia and hypertension [1]. Diabetes is a prime cause of afflictions such as blindness, limb amputation and other diseases such as kidney failure and cardiovascular diseases including stroke and heart attack [2]. Approximately 463 million people are affecting with diabetes worldwide and this number will increase to 700 million by 2045. Type-2 diabetes comprises 90% around the world and more prevalent when compared with type-1 diabetes [3]. Management of diabetes mellitus normally involves diet, exercise and chemotherapy. The conventional pharmacological treatments are associated with many adverse side effects and high rates of secondary failure which lead to an increasing demand for natural products with anti-diabetic activity and lesser side effects [4]. Thus, it is important to look for more potent antidiabetic agents preferably from dietary sources, which should be cost effective and have lesser or no side effects. Many experimental studies have elucidated that flavonoids, terpenoids and other secondary metabolites of plant possess hypoglycemic effects in different experimental models [5] Hence trends on analysing the hypoglycemic effect of herbal and natural compounds gained great interest due to its abundance in plants and efficacy. β- Caryophyllene is a naturally occurring sequiterpene found in cannabis as well as a variety of culinary herbs and spices. This terpene may be found in black pepper, cloves, cinnamon, hops, rosemary, and hemp. It has a wide range of biological actions, including antioxidant, anti-inflammatory, and anti-lipidemic properties [6]. It may aid in the reduction of cholesterol, the relief of pain and anxiety, and the treatment of seizures. Being a anti-inflammatory agent β-Caryophyllene protects against oxidative stress, it might be a beneficial preventative medication for a variety of medical problems such as liver diseases, renal diseases, liver and gastrointestinal illnesses, immunological and neurological diseases [6, 7, 8, 9]. In streptozotocin (STZ)-induced diabetic mice, chronic oral treatment of β-Caryophyllene decreases glyceamia, depressive-like behaviour, and neuropathic pain [10]. Furthermore, it has recently been discovered that β-caryophyllene efficiently protects -cells by relieving hyperglycemia by boosting insulin release, as well as reducing oxidative stress and inflammation in the pancreatic tissue of diabetic rats [11] However, the effect of β-Caryophyllene on glucose uptake, oxidation is still unclear. Hence, the present study was designed to explore the effect of the antidiabetic effect of β-Caryophyllene in high fat diet- induced type-2 diabetes.

## Methodology:

### Animals:

In this study, we used 150-180 day old Wistar strain healthy adult male albino rats. The Institutional Animal Ethics committee (IAEC No: 007/2019, dated 04/11/2019) at Meenakshi Medical College and Research Institute, MAHER, Enathur, Kanchipuram, Tamil Nadu- 631552, India, approved their treatment in line with national rules and protocols. Animals were housed at a constant temperature (21± 2°C) and humidity (65 ± 5%), with a 12 hour light and 12 hour dark cycle, and fed a normal pelleted food (Lipton India, Mumbai, India), with clean drinking water supplied ad libitum.

### Induction of Type-2 diabetes:

Rats were fed a high fat diet comprising 2% cholesterol, 1% cholic acid, 30% coconut oil, 67 percent regular rat feed, and 25% fructose through drinking water to make them diabetic (type-2) for 60 days [12]. Fasting blood glucose levels were measured after 60 days, and animals with blood glucose levels of >120 mg/dl were chosen for the experiment. The high-fat diet and sugar feeding were maintained until the study's conclusion. Normal pelleted rat feed was provided to control rats, and water was freely available.

### Experimental design:

The following experimental design was framed and accordingly the rats were subjected to treatment for a period of one month. Healthy adult male Wistar rats were divided into the following groups of 6 rats each.

Group I: Control (Normal rats).

Group II: Rats were made diabetic (type-2) after feeding high fat diet & fructose through drinking water (30%) for 60 days.

Group III: Type-2 diabetic rats treated orally with β-caryophyllene (200 mg/kg b.wt/day) for 30 days.

Group IV: Type-2 diabetic rats treated orally with metformin (50 mg/kg, b.wt/day) for 30 days.

Group V: Control rats administered orally with β-caryophyllene (200 mg/kg b.wt/day) for 30 days.Two days before they were killed, control and experimental rats were given an oral glucose tolerance test (OGTT) and an insulin tolerance test (ITT). Blood was collected after 30 days, and the animals were perfused with physiological saline while anaesthetized with sodium thiopentone (40 mg/kg b.wt), and skeletal muscle was torn out to assess various parameters.

### Fasting blood glucose (FBG):

After overnight fasting, blood glucose was measured using On-Call Plus blood glucose test strips (ACON Laboratories Inc., USA). Blood was obtained by pricking the rat's tail tip, and the results were reported in mg/dl.

### Serum insulin:

This test was carried out using a Crystal ChemInc ultrasensitive rat insulin ELISA kit (Illinois, USA). The detection range is 0.1 to 64 ng/ml. Insulin antibody had a 100 percentage cross-reactivity with rat insulin. There was a 10.0 percent intra-assay coefficient of variance and the results are given in nanograms per millilitre.

### Homeostasis Model Assessment for Insulin Resistance (HOMA-IR):

HOMA-IR was calculated using the formula (fasting blood glucose X fasting serum insulin/405) based on the method of Matthews et al. [13].

### Oral Glucose Tolerance Test (OGTT):

Animals were fasted overnight for the oral glucose tolerance test, and blood glucose was measured using On-Call Plus blood glucose test strips at various time intervals (60, 120, and 180 minutes) after receiving the oral glucose load (10 ml/kg; 50% w/v). The blood glucose level before the glucose load is referred to as the 0 minute value. The results are given in milli grams per deciliter (mg/dl).

### Serum lipid profile:

Assay kits from Spin react, Spain, were used to measure serum cholesterol (CHO), triglycerides (TG), low-density lipoproteins(LDL), and high-density lipoproteins (HDL). The results are expressed in mg/dl.

### Statistical analysis:

Using computer-based software, the data were analysed using one-way analysis of variance (ANOVA) and Duncan's multiple range test to determine the significance of individual differences between the control and treatment groups (Graph Pad Prism version 5). The significance of Duncan's test was determined at the level of P<0.05.

## Results and Discussion:

Type 2 diabetes is a complex, heterogeneous and polygenic disease that is becoming an important cause of morbidity and mortality. Body's resistance to insulin is one of the important factors contribute to the hyperglycemia in type-2 diabetes [14]. Many experimental studies have explored that rats feeding with high-fat diet (HFD) develops insulin resistance and mimicking the clinical features and pathogenesis of type 2 diabetes mellitus as occurred in human [15] Hence in our study, we used high fat-diet for induction of type-2 diabetes in rats. This model provides a perfect platform for screening of antidiabetic agent, as interest on investigation of natural remedy is at the peak due to the crooked features of the regular therapies for diabetes. Our study evidenced the antidiabetic efficacy of β-Caryophyllene by assessing various biological parameters, glucose homeostasis and lipid profile highly noticeable during diabetes. Hyperglycemia is the main characteristic of Diabetes mellitus. Blood glucose control is an important tool in preventing or delaying the complications of diabetes. We observed a significant increase in blood glucose and insulin levels as a result of insulin resistance in target tissue induced by high fat diet in diabetic rats. Supplementation of β-Caryophyllene to diabetic rats resulted in reduced blood glucose and restored insulin levels is shown (Figure 1A & 1B - see PDF) [11].

Moreover, the degree of insulin resistance and hyper insulinemia was justified from HOMA-IR. High fat diet- induced diabetic rats showed significantly higher values of HOMA-IR, whereas β-Caryophyllene treatment resulted in a pronounced decrease in the HOMA-IR (Figure.2-see PDF), elucidates the efficacy of β-Caryophyllene in restoring insulin sensitivity. Oral glucose tolerance test (OGTT) is the most common and highly sensitive for early abnormalities in the regulation of glucose than fasting plasma glucose and HbA1C [16].The data obtained in OGTT (Figure.3A-see PDF) also confirms the anti-hyperglycemic potential of β-Caryophyllene. In the present study, the diabetic rats showed elevated levels of glucose even after 2h. Whereas, in β-Caryophyllene treated diabetic rats, glucose levels were returned to fasting values after 2h. Our results revealed that β-Caryophyllene treatment has the ability to normalize the insulin secretion and thereby, reduces the elevated blood glucose levels. Figure.3B represents the effect of β-Caryophyllene on insulin tolerance test. When control rats were given an insulin load, it showed a substantial reduction, reaching a minimum in 30 minutes and returning to near normal range in 60 minutes. Insulin treatment to diabetic rats causes a delayed decrease in blood glucose levels, which only reaches a minimum after 60 minutes, indicating poor insulin tolerance. As with the conventional medication metformin, caryophyllene therapy increases insulin tolerance in diabetic rats.

Dyslipidemia is another consequence, occurred during diabetes plays a key role in the diabetes pathogenesis and its complications. High fat diet and fructose-induced diabetic rats showed elevated levels of triglycerides and cholesterol which may be the result of increased de novo synthesis of lipids [17]. As well as considerable increase in LDL-C levels and a significant decrease in the levels of HDL-C were also observed in diabetic rats which is may be due to impairment in the LDL receptor activity and exchange of triglycerides between VLDL and HDL for cholesterol esters [18]. β- Caryophyllene treatment considerably restored the altered levels of lipids near to normal level as that of standard drug (Figure 4A, 4B, 4C & 4D-see PDF). It may probably the result of its influence on lipid metabolism and its transport. The present data evidenced the hypolipidemic activity of β-Caryophyllene.

## Conclusion:

The results of the present study elucidated that administration of β- Caryophyllene significantly improved glycemic status through improving dyslipidemia in a similar way as metformin thereby improved insulin signalling in diabetic rats. Hence, β-sCaryophyllene can be used as one of the potential drugs for the management of type-2 diabetes and need further studies to ascertain its potential.
